# Results of endoscopic biliary drainage in patients with malignant hilar stricture

**DOI:** 10.1016/j.clinsp.2022.100153

**Published:** 2023-01-20

**Authors:** Bruno Costa Martins, Caio A. Perez, Jennifer N. Ruas, Luiza H. Bento, Ernesto Q. Mendonça, Gustavo A. de Paulo, Ricardo S. Uemura, Sebastian N. Geiger, Marcelo Simas de Lima, José Jukemura, Ulysses Ribeiro, Fauze Maluf-Filho

**Affiliations:** a Division of Endoscopy, Instituto do Câncer do Estado de São Paulo, Department of Gastroenterology of Universidade de São Paulo, São Paulo, SP, Brazil; b Division of Gastrointestinal Surgery, Instituto do Câncer do Estado de São Paulo. Department of Gastroenterology of Universidade de São Paulo, São Paulo, SP, Brazil

**Keywords:** Malignant hilar biliary obstruction, Therapeutic endoscopy, stents

## Abstract

In Malignant Hilar Biliary Stricture (MHBS) palliative biliary drainage is a frequent strategy, improving the quality of life, reducing pruritus, loss of appetite and relieving cholangitis. The endoscopic approach is an effective, although challenging procedure. This study aimed to evaluate technical and clinical success rates of biliary drainage by ERCP. This is a retrospective study including all patients with MHBS referred to Instituto do Cancer do Hospital de São Paulo (ICESP) submitted to biliary drainage by ERCP, between January 2010 and December 2017. Multivariable logistic regression was performed to evaluate predictors of clinical failure, as total bilirubin levels, Bismuth classification, number of hepatic sectors drained and presence of cholangitis. In total, 82 patients presenting unresectable MHBS were included in this study. 58.5% female and 41.5% male, with a mean age of 60±13 years. Bismuth classification grades II, IIIA, IIIB and IV were noted in 23.2%, 15.9%, 14.6% and 46.3%, respectively. Technical and clinical success was achieved in 92.7% and 53.7% respectively. At multivariable logistic-regression analyses, Bismuth IV strictures were related to higher clinical failure rates when compared to other strictures levels, with an Odds Ratio of 5.8 (95% CI 1.28‒20.88). In conclusion, endoscopic biliary drainage for malignant hilar biliary stricture had a high technical success but suboptimal clinical success rate. Proximal strictures (Bismuth IV) were associated with poor drainage outcomes.

## Highlights

• Malignant hilar biliary stricture had a high technical success rate but suboptimal clinical success rate.
• At multivariable logistic-regression analyses, Bismuth IV strictures were related to higher clinical failure rates when compared to other strictures levels.
• There was a trend towards higher failure rates with increased levels of pre-drainage bilirubin level, although not statistically significant.

## Introduction

Malignant Hilar Biliary Stricture (MHBS) is caused by primary hepatobiliary neoplasms, as well as metastatic progression of extrahepatic neoplasms. Given the absence of early symptoms, patients usually present at an advanced stage of the disease, making curative treatment unlikely in most cases.^1^, ^2^, ^3^

In this scenario, palliative biliary drainage is a frequent strategy, improving the quality of life, reducing pruritus, loss of appetite and relieving cholangitis.^4^

The endoscopic approach to drain bile ducts with placement of plastic or metal stents by Endoscopic Retrograde Cholangiopancreatography (ERCP) has been shown to be an effective strategy over the last decades. However, it is noteworthy that it is usually a challenging and complex procedure, especially when there is more proximal involvement of the hepatobiliary tract.^5^^,^^6^

Therefore, this study aimed to evaluate the technical and clinical success rates of biliary drainage by ERCP in patients with primary or secondary MHBS and to analyze predictors factors of failure.

### Patients and methods

This is a retrospective study including all patients with MHBS referred to the endoscopic service of the Instituto do Cancer do Hospital de São Paulo (ICESP) submitted to biliary drainage by ERCP, between January 2010 and December 2017. Follow-up and survival data were obtained from medical records. The study was approved by Institution's ethics committee. Were included patients presenting unresectable MHBS classified as Bismuth II, III or IV with indication of biliary drainage, submitted to ERCP between January 2010 and December 2017 with complete data in medical records. Were excluded patients under 18 years old, patients presenting unresectable MHBS not submitted to endoscopic biliary drainage, patients with incomplete data in medical records, patients with resectable MHSB and with MHBS classified as Bismuth I.

All patients admitted to our hospital must have a confirmed malignant disease. Confirmation of malignancy in this group of patients with hilar strictures was made by histopathology obtained from ERCP, EUS, or CT-guided biopsy. In cases where histopathology wasn't possible to obtain, or if it was inconclusive, a strong suspicious based on clinical presentation, CT and ERCP findings, was also acceptable. In these cases, the diagnosis must had been subsequently confirmed by the clinical course of the disease.

The indications for ERCP were cholangitis, previous biliary stent dysfunction, or palliation of malignant stenosis. The decision regarding the type of stent used, as well as unilateral or bilateral drainage, was made according to the indication and the technical aspects of the procedure observed in each case. Briefly, when bilateral drainage was attempted, two or more plastic stents were introduced. After this first endoscopic session, the plastic stents were replaced by metallic stents 60‒90 days later.

Technical success was defined as the placement of plastic or metallic stents above the hilar stricture, draining at least the hepatic lobe with the major dilation on previous CT findings and the segments filled with contrast during ERCP. Patients with technical failure were referred to transparietohepatic drainage. The clinical success was defined as a decrease in bilirubin levels of more than 50% of the pre-treatment value, measured 2-weeks after the procedure as defined in Tokyo criteria 2014.^7^ The predictors of clinical failure included for analysis were pre-drainage bilirubin levels, presence of cholangitis, stricture level according to Bismuth classification and the number of drained hepatic segments by ERCP.

Cholangitis was diagnosed according to Tokyo Guidelines 2018: evidence of systemic inflammation (fever, elevated C-reactive protein or Leukocytosis), signs of cholestasis (jaundice and/or laboratorial abnormal liver function tests) and biliary dilation or evidence of the etiology on imaging studies were present.^8^

Bismuth type was determined based on radiologic findings (magnetic resonance cholangiography, computed tomography and/or direct cholangiography), and only patients with Bismuth ≥ II were included.

The hepatic drained segments were calculated dividing liver parenchyma into three sectors: left (segments II, III and IV); right anterior (segments V and VIII); right posterior (segments VI and VII).

### Statistical analysis

For analysis of predictors of clinical failure, multivariable logistic-regression analysis was performed to evaluate the effect of total bilirubin levels, Bismuth classification, number of hepatic sectors drained and presence of cholangitis. These four variables were included in the regression models, using one approach: analyzing the effects of the four variables separately. The quality of the model was given using Cox and Snell's R^2^ and Nagelkerke's test. Logistic-regression coefficients (B) were transformed to odds ratios and 95% confidence intervals using standard methods. Pearson's Chi squared test was performed to compare clinical success in primary biliary tract neoplasm (Cholangiocarcinoma and gallbladder adenocarcinoma) with neoplastic extrinsic compression

## Results

In total, 82 patients presenting unresectable MHBS were included in this study, 58.5% female and 41.5% male, with a mean age of 60 ± 13 years. Bismuth classification grades II, IIIA, IIIB, and IV were noted in 23.2%, 15.9%, 14.6% and 46.3%, respectively. The indication of ERCP was palliative drainage in 56.1%, cholangitis in 29.3%, previous stent obstruction in 14.4% and malignant fistula due to cholangiocarcinoma in one case (1.2%). From them, 39% had a previous stent, 34.1% had plastic stent and 4.9% had metallic stent. The mean direct and total bilirubin levels were 8.2 mg/dL and 11.56 mg/dL, respectively.

Technical success was achieved in 92.7% of patients. Biliary access was achieved 47.4% through the papilla, 10.5% through fistulotomy and 42.1% by previous papillotomy. Regarding patients with technical failure, 5 (6.1%) were referred for transparietohepatic drainage and 1 (1.2%) underwent a new endoscopic procedure with cholangioscopy.

Plastic stents were initially placed in 68.3% of patients and metallic stents in 24.3% (3.6% partially covered, 1.2% totally covered and 19.5% uncovered). Most of the patients had more than one hepatic segment drained (71.9%). In 20.7%, just one hepatic lobe or segment was drained, and 7.31% patients had unsuccessful drainage. Clinical success rate was 53.7%. Adverse events after ERCP included cholangitis (12.2%), pancreatitis (4.9%), cholecystitis (1.2%) and stent obstruction (14.6%) (Table 1).

**Table 1:** Baseline characteristics of patients who underwent endoscopic biliary drainage with malignant hilar stricture.

	Overall (%)
Gender	
Female	48 (58.5)
Male	34 (41.5)
Biliary strictures	
Bismuth II	19 (23.2)
Bismuth IIIa	13 (15.8)
Bismuth IIIb	12 (14.6)
Bismuth IV	38 (46.3)
Indications	
Cholangitis	24 (29.3)
Palliative drainage	46 (56.1)
Stent dysfunction	11 (14.4)
Biliary malignant fistula	1 (1.2)
Etiology of MBHS	
Cholangiocarcinoma	27 (32.9)
Colorectal lymph node metastasis	20 (24.4)
Other lymph node metastasis	12 (14.6)
Gallbladder carcer	12 (14.6)
Pancreatic cancer	6 (7.3)
Lymphoma	2 (2.4)
Hepatocarcinoma	1 (1.2)
Previous stent	
None	50 (61)
Plastic	28 (34.1)
Metallic	4 (4.9)
Serum Bilirubin (mg/dL)	
Total	11.56 (23.4)
Direct	8.2 (11.8)
Technical Success	76 (92.7)
Bismuth II	19 (100)
Bismuth IIIa	12 (92.3)
Bismuth IIIb	12 (100)
Bismuth IV	33 (86.84)
Biliary access	
Transpapillar	36 (47.4)
Fistulotomia	8 (10.5)
Previous papillotomy	32 (42.1)
Clinical Success	44 (53.7)
Bismuth II	14 (73.7)
Bismuth IIIa	8 (66.7)
Bismuth IIIb	7 (58.3)
Bismuth IV	14 (42.4)
Stent type	
Metallic PC	3 (3.6)
TC	1 (1.2)
UC	16 (19.5)
Plastic	56 (68.3)
Drained area	
Unsuccessful drainage	6 (7.31)
1 sector	17 (20.7)
> 1sector	59 (71.9)
Adverse Events	
Cholangitis	10 (12.2)
Pancreatitis	4 (4.9)
Cholecystitis	1 (1.2)
Stent Obstruction	12 (14.6)

Comparing clinical success of patients with primary biliary tract neoplasm (cholangiocarcinoma and gallbladder cancer) versus patients with neoplastic extrinsic compression (lymph node metastasis) we found no difference between groups (p = 0.928).

At multivariable logistic-regression analyses, the only prognostic factor related to clinical success was the level of hilar stricture. Bismuth IV strictures were related to higher clinical failure rates when compared to other strictures levels, p = 0.021 with an odds ratio of 5.18 (95% CI 1.28‒20.88). There was a trend towards higher failure rates with increased levels of pre-drainage bilirubin level, although not statistically significant (OR = 1.07; 95% CI 0.99‒1.15; p = 0.052). Cholangitis and multi lobar drainage were not predictors of clinical failure (Table 2).

**Table 2:** Multivariable logistic-regression analysis of factors predictors of clinical failure.

Variable	Odds ratio (95% CI)	p-value
Biliary strictures		
Others	1.0	
Bismuth IV	5.18 (1.28‒20.88)	0.021
Sector		
1 sector	1.0	
> 1 sector	2.31 (0.62‒8.59)	0.213
Cholangitis		
Absence	1.0	
Presence	4.15 (0.78‒22.04)	0.095
Total Bilirubin levels	1.07 (0.99‒1.15)	0.052

The model included all factors as covariables. CI, Confidence Interval. p-value < 0,05 was considered significant.

## Discussion

ERCP with placement of biliary plastic or metal stents is widely used for palliative therapy of biliary obstruction caused by malignant hilar neoplasms to improve and maintain quality of life in patients with advanced disease who are not candidates for surgical treatment.^9^

The clinical success rate obtained with endoscopic drainage is attributed to several variables, such as the extension of disease, type of stent, bilateral versus unilateral drainage of the liver parenchyma and the endoscopic technique used.^10^, ^11^, ^12^

In the present study, we observed a clinical success of 53.7%, corroborating the fact that the endoscopic approach to drainage of bile ducts in hilar neoplasms is a challenging scenario. A multicenter study evaluating risk factors for drainage failure for resectable malignant hilar tumors including only plastic stents, showed a technical failure rate of 6%, similar to 7.3% found in our study.^13^ In multivariate analysis the authors found that a pre-drainage total bilirubin level above 8.8 mg/dL and the proximal extent of bile duct obstruction were independent predictors of clinical failure.^13^

In our study, we observed that Bismuth IV hilar obstruction was associated with higher rates of clinical failure and, although not statistically significant, there was a trend towards higher failure rates with increased pre-drainage total bilirubin levels. We cannot exclude a type II error (beta error) here. A larger cohort might have shown the effect of pre-drainage bilirubin level. Our results are in line with the results of the previous mentioned study of Wiggers et al.^13^

There is an intense debate in the literature if bilateral is superior to unilateral drainage in MHBS. Although several studies and meta-analyses showed no superiority of bilateral versus unilateral drainage,^14^, ^15^, ^16^ one randomized controlled trial showed that bilateral drainage is related to better clinical success rates and a reduced incidence of stent dysfunction compared with unilateral stenting in MHBO patients.^17^ In our study the quantity of hepatic sectors drained was not associated with therapeutic failure. This might be explained by the pre-operative strategy adopted in our routine. Before endoscopic procedures, all patients with MHBS have their RNM and CT scans carefully analyzed, considering the amount of viable liver parenchyma and the segments mainly involved by biliary obstruction (major biliary dilation) and a roadmap strategy was adopted. More than one hepatic segment drainage was always intended in cases of Bismuth IIIa and IV, while unilateral drainage was an acceptable approach in cases of Bismuth II and IIIb. In our study, clinical success rate was 40% in Bismuth IIIa and IV when only one sector was drained. When more than one sector was drained, this rate rose to 53.5% (no difference). In the literature, a volume of hepatic liver parenchyma of less than 50% (which is approximately compatible with drainage of only 1 sector in our study) is related with therapeutic failure.^5^^,^^18^^,^^19^

This study has some limitations. First, this is a retrospective study with inherited limitations of its design. Survival analysis was not performed, which is an important clinical outcome to be considered in this population. Pre-drainage volume assessment was not routinely performed, which could impact favorably in patient's outcomes if a strategy to drain more than 50% of liver parenchyma was adopted.^18^^,^^20^ However, routine application of hepatic volume is difficult to apply. In this sense, the strategy of numbering the quantity of liver segments that are drained is more practical.

Despite those limitations, our findings support that patients with Bismuth IV strictures should be referred to other strategies approaches, such as endoscopic ultrasonography-guided, percutaneous transhepatic biliary drainage or the combination of two or more routes.^21^^,^^22^

In conclusion, our casuistic demonstrates that endoscopic biliary drainage for malignant hilar biliary stricture had a high technical success but suboptimal clinical success rate, probably due to high percentage of proximal strictures (Bismuth IV) which are seen to be associated with poor drainage outcomes.

## Conflicts of interest

The authors declare no conflicts of interest.
